# Incidence, Risk Factors, and Survival of Bone Metastases and Skeletal-Related Events in Melanoma Patients: A Systematic Review and Quality Assessment of 29 Studies

**DOI:** 10.1016/j.jbo.2024.100603

**Published:** 2024-04-22

**Authors:** Michelle R. Shimizu, Olaf N. van de Langerijt, Daniel Torres, Tom de Groot, Olivier Q. Groot

**Affiliations:** a Loyola University Stritch School of Medicine, Maywood, IL, USA; b Department of Orthopedic Surgery, University Medical Center Utrecht, Utrecht, the Netherlands; c Department of Orthopedic Surgery, University Medical Center Groningen, Groningen, the Netherlands

**Keywords:** Bone metastases, Melanoma, Incidence, Risk factors, Survival, Skeletal-related events

## Abstract

**Background:**

Skeletal metastases make up 17% of all metastases from advanced-stage melanoma. Bone metastases are associated with increased morbidity and mortality and decreased quality of life due to their association with skeletal-related events (SREs), including pathological fracture, spinal cord compression, hypercalcemia, radiotherapy, and surgery. The study aimed to determine the incidence of bone metastases and SREs in melanoma, identify possible risk factors for the development of bone metastases and SREs, and investigate survival rates in this patient population.

**Methods:**

A computer-based literature search was conducted using Pubmed, Embase, and Cochrane Central Register of Controlled Trials up to July 2023. The Newcastle-Ottawa Quality Assessment Scale (NOS) was utilized for quality assessment. Study characteristics, patient information, risk factors for developing bone metastases and SREs, and characteristics for survival were recorded.

**Results:**

We included 29 studies. The average bone metastasis-free interval ranged from four to 72 months. Incidence of bone metastases varied from 2 % to 49 % across 14 studies. 69 % (20/29) of studies described the location of bone metastases, with 24 % (7/29) focusing solely on spinal metastases. In one study, 129 SREs were recorded in 71 % (59/83) of the patient cohort, with various manifestations. The use of bone-directed agents was independently associated with lower risk of SREs. Survival after detection of bone metastasis ranged from three to 13 months. Factors associated with survival included clinical, tumor-related, and treatment features.

**Conclusion:**

This review highlights the notable prevalence and risk factors of developing bone metastases and subsequent SREs in patients with melanoma. The surge in bone metastases poses a challenge in complication management, given the high prevalence of SREs. While this study offers a comprehensive overview of the incidence, risk factors, and outcomes associated with bone metastases and SREs in melanoma patients that may guide patient and physician decision-making, a notable gap lies in the limited availability of high-quality data and the heterogeneous design of the existing literature. Future research should address predictive factors for bone metastases and SREs in melanoma to facilitate patient and physician decision-making and ultimately improve outcomes in this patient population.

## Highlights

• A considerable amount of melanoma patients suffers from bone metastases.
• Bone metastases increase morbidity and mortality through skeletal-related events.
• Bone-directed agents lower skeletal-related event risk in melanoma bone metastases.
• There are limited studies on risk factors associated with skeletal-related events.
• Bone metastasis survival is linked to clinical, tumor, and treatment factors.

## Graphical abstract

**Figure:**
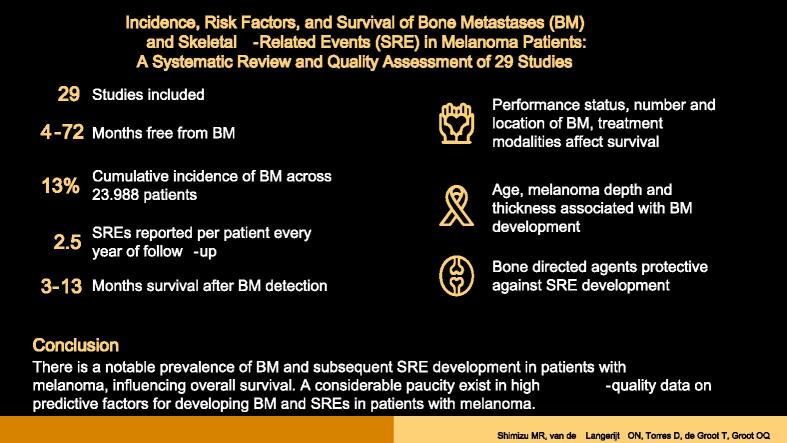

## 1 Introduction

Melanoma was the fifth most common cancer diagnosis in the United States in 2021, accounting for 5.6 % of all cancer diagnoses and an estimated 106,000 new cases annually [1]. Melanoma’s aggressive progression and development into bone metastasis makes the disease accountable for most skin cancer-related deaths [2]. It is estimated that three to seven percent of patients with melanoma develop bone metastasis, representing up to 17 % of the metastases from metastatic melanoma [3], [4], [5]. In recent years, advancements in melanoma diagnosis and treatment, such as multi-modal therapies including novel immunotherapies and targeted drugs, as well as enhanced imaging methods, have improved survival but have simultaneously led to a rising incidence of bone metastases [6].

Bone metastases are associated with increased morbidity and mortality and decreased quality of life due to its association with skeletal-related events (SREs), which include pathological fracture, spinal cord compression, hypercalcemia, radiotherapy, and surgery [7]. The high prevalence of melanoma makes the burden of bone metastasis and subsequent SREs considerable. A recent retrospective study reported that melanoma patients with bone metastasis suffered an SREs incidence rate of 66 %, of which radiotherapy was the most common SRE, with an incidence rate of 39 % [4]. Despite the high prevalence of bone metastases and SREs in patients with melanoma, information on the overall incidence, risk factors, and prognosis is limited. A better understanding of the disease burden of bone metastases and SREs in patients with melanoma may guide healthcare management, improve the quality of care, and help clinicians and patients in management decisions to minimize the detrimental effects of skeletal metastases and SREs.

To our knowledge, only a handful of studies specifically report on bone metastases and SREs of patients with melanoma, and no systematic reviews have been conducted on this topic. Therefore, this systematic review aims to report the incidence, risk factors of developing bone metastases, and subsequent SREs in patients with melanoma. Our study offers the first overview of the scale of both bone metastases and SREs that can be used to guide the management of bone metastases in patients with metastatic melanoma to achieve the best possible outcome.

## 2 Methods

### 2.1 Protocol and registry

This systematic review was performed according to Preferred Reporting Items for Systematic Reviews and Meta-Analyses (PRISMA) guidelines [8]. We registered the protocol online in PROSPERO with CRD42023410881.

### 2.2 Search strategy

Three independent reviewers (M.R.S., O.N.L., D.T.) performed a systematic literature search using PubMed, EMBASE (OvidSP), and Cochrane Central Register of Controlled Trials for studies published from their inception to July 23, 2023. Boolean searches were performed using Medical Subject Headings (MeSH) for PubMed and Cochrane, Emtree for EMBASE, or keywords and search terms combining “melanoma” and “bone metastases” (Appendix A).

### 2.3 Eligibility criteria

**Fig. 1:**
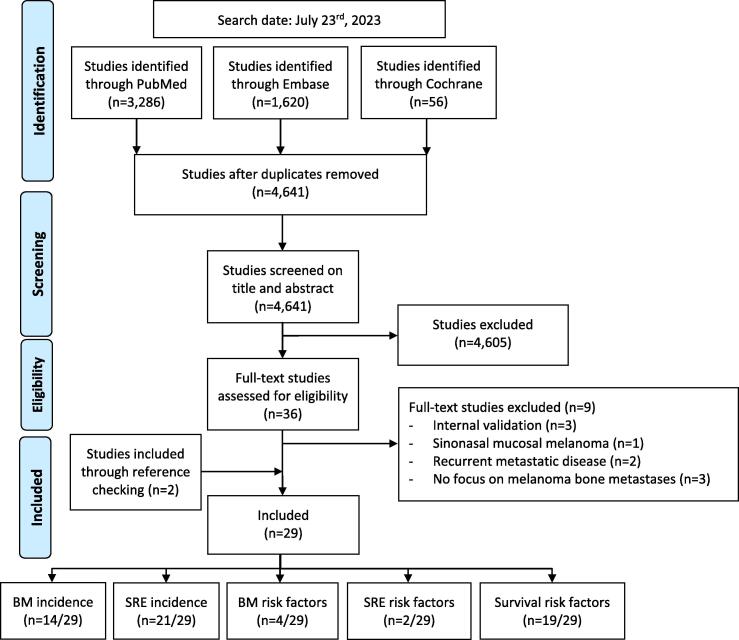
Flowchart of included studies (n = 29).

All titles and abstracts from the literature search were exported into Mendeley and uploaded to Rayyan [9]. A series of consecutive stages were performed for study selection, including duplicate identification, screening of titles and abstracts, and full article review in accordance with the eligibility criteria by the same three independent reviewers. All human studies in English related to melanoma, bone metastases (including spinal metastases), and SREs were included. Studies were excluded if they (1) were case series, letters, comments, editorials, conferences, guidelines, abstracts, supplements, technique papers, systematic reviews, or regular review articles; (2) included fewer than ten patients with bone metastases from melanoma; (3) focused on metastatic uveal melanoma or sinonasal mucosal melanoma; (4) did not prioritize melanoma as the primary cancer, focused on visceral metastases or oligometastatic disease, or prioritized recurrent disease; or (5) were clinical trials focusing on metastatic melanoma treatment unless median survival was explicitly mentioned. Metastatic uveal and sinonasal mucosal melanoma patients were excluded given their distinct biological and clinical features compared to cutaneous melanoma that contribute to significantly different prognosis and overall survival as demonstrated in previous studies [10], [11]. All included studies were manually referenced and reviewed for any missed studies. Disagreements or uncertainties were resolved through discussion with an experienced fourth reviewer (O.Q.G.; Fig. 1).

### 2.4 Quality assessment

The quality of the included studies was independently assessed by two reviewers (M.R.S., O.N.L.) using the Newcastle-Ottawa Quality Assessment Scale (NOS) [12]. The NOS was developed to determine the quality of cohort- and case-control studies using a “star system” in which a study is evaluated on selection, comparability, and exposure/outcome of interest of the study group. Study ratings ranged from zero to nine: studies with more than six stars were deemed to have a “low risk of bias”, those with three to five stars an “unclear risk of bias”, and those with less than two stars a “high risk of bias” [12]. Any disparities were discussed with a third reviewer (O.Q.G.) present to reach a final consensus.

### 2.5 Data extraction

The following data were extracted from each study independently by two investigators (M.R.S. and O.N.L.) — (1) study characteristics: first author, year of publication, study country, study type, and study period; (2) patient information: demographics, tumor characteristics, and bone metastases; (3) statistical analysis of risk factors for developing melanoma bone metastases and SREs; (4) statistical analysis of prognostic characteristics influencing survival in patients with melanoma bone metastases; and (5) data on bone metastases diagnosis, SREs development, therapy, and prognostic outcomes, when provided. Data not mentioned in the text of the studies but available on graphs or subgroup analysis were extrapolated and included in this systematic review.

### 2.6 Study inclusion and quality assessment

In total, 4,641 studies were screened after removing all duplicates. After screening the title and abstract, 4,605 studies were excluded, leaving 36 full-text studies. After assessing eligibility based on the selection criteria, the final set comprised 29 studies (Appendix B) [4], [5], [13], [14], [15], [16], [17], [18], [19], [20], [21], [22], [23], [24], [25], [26], [27], [28], [29], [30], [31], [32], [33], [34], [35], [36], [37], [38], [39]. Study quality assessment using NOS demonstrated that 66 % (19/29) of studies had a “low risk of bias” with scores above six, and 34 % (10/29) of studies had an “unclear risk of bias” with a NOS score of five. An “unclear risk of bias” was mainly due to the low comparability scores, as most studies involved only one cohort. All 29 studies were included for data extraction as none had a score lower than five, and thus, none were considered to have a “high risk of bias”. (Appendix C, D).

### 2.7 Study characteristics

Except for one prospective single-center study, all 29 investigations were retrospective. Of the 28 retrospective cohorts, 79 % (22/28) were either single-center or two-center studies. Study periods ranged from three to 40 years between 1955 and 2019. Median follow-up time ranged from three to 76 months, with one study recording a minimum follow-up of 60 months [37]. 97 % (28/29) of studies focused solely on patients with melanoma as the primary tumor, and 59 % (17/29) of studies included only patients with bone metastatic melanoma. The number of patients enrolled in the studies ranged from 11 to 7,010. Of the 23,998 patients included in the 29 studies, 13 % (3,130/23,998) had a recorded bone metastatic melanoma. Male patients comprised 56 % of the study cohorts while female patients comprised 44 %. The median age of melanoma diagnosis ranged from 42 to 67 (Table 1).

**Table 1:** Cohort characteristics of all included studies with melanoma patients (n = 29).

First author; year	Country	Study type	Years	Total Patients (N)	Total BMM (N (%))	Mean/ median Age (yr)	M/F	Stage distribution (N (%))	BRAF; NRAS(N (%))	T1	Overall survival^a^(mo)	Follow up (mo)
Abdel-Rahman O;2018	Egypt	Retrospective,population-based	2010–2013	2.691	572 (21)	−	−	−	−	−	−	−
Akslen L; 1987	Norway	Retrospective, single-center autopsy	1955–1984	62	37 (49)	52	39/23	I 44 (71);others unknown	−	−	−	−
Angela Y; 2019	Germany	Retrospective,multi-center	−	29	29 (100)	61	20/9	−	BRAF 17 (59)	−	−	20^b^
Berberich C; 2023	Germany	Retrospective,single-center	2012–2018	93	−	61	57/36	−	−	−	−	3^b^
Bhanot K; 2022	Canada	Retrospective,population-based	2006–2016	2.646	91 (3)	63	1.452/1.194	−	−	−	−	−
Bostel T; 2016	Germany	Retrospective,two-center	2003–2013	41	41 (100)	66	24/17	IV 41 (100)	−	−	−	4^c^; 6^b^
Brountzos E; 2001	Greece	Retrospective,single-center	1985–2000	293	28 (10)	50	−	IV 293 (100)	−	BM detection	5^b^	−
Brown L; 2023	Australia	Retrospective,two-center	2012–2017	264	24 (12)	−	176/88	IV 24 (100)	BRAF 39 (19)	−	−	Minimum 60
Colman M; 2014	USA	Retrospective,single-center	1998–2010	130	130 (100)	−	86/44	IV 130 (100)	−	BM detection	6^b^	−
DeBoer D; 1996	USA	Retrospective,single-center	1962–1992	283	14 (5)	−	−	IV 14 (100)	−	BM detection	13^c^	−
Do-ldson W; 1993	USA	Retrospective,single-center	1986–1991	15	15 (100)	42	11/4	−	−	BM detection	6^c^	−
Fon G; 1981	USA	Retrospective,single-center	1970–1979	50	50 (100)	45	33/17	IV 50 (100)	−	BM detection	6^c^	4^c^
Gassenmaier M; 2019	Germany	Retrospective,single-center	1975–2015	3.139	431 (30)	57	834/623	−	−	−	−	50^b^
Gerszten P; 2005	USA	Prospective,single-center	2002–2005	28	28 (100)	47	17/11	−	−	−	−	13^b^
Gokaslan Z; 2000	USA	Retrospective,single-center	1984–1994	133	133 (100)	47	82/51	−	−	BM detection	4^b^	−
Huang K; 2007	Taiwan	Retrospective,two-center	1983–2006	11	11 (100)	62	6/5	−	−	BM detection	6^c^	−
Huttenlocher S; 2014	Saudi Arabia	Retrospective	−	27	27 (100)	−	18/19	−	−	Radiotherapy	−	Up to 12
Krygier J; 2014	USA	Retrospective,single-center	1990–2006	37	37 (100)	51	21/16	−	−	Surgery	−	6^c^
Manavola F; 2020	Italy	Retrospective,multi-center	1984–2019	305	305 (100)	56	193/112	−	BRAF 168 (59) NRAS 21 (36)	BM detection	11^b^	−
Rate W; 1988	USA	Retrospective,two-center	1980–1987	77	26 (34)	−	−	−	−	Radiotherapy	−	−
Sellin J; 2015	USA	Retrospective,single-center	1993–2012	64	64 (100)	49	44/20	−	−	Surgery	6^b^	3^b^
Shankar G; 2017	USA	Retrospective,single-center	2012–2015	18	18 (100)	−	−	−	BRAF 6 (46)	Intervention	−	3^b^
Spiegel D; 1995	USA	Retrospective,single-center	1970–1991	7.010	114 (2)	44	−	−	−	BM detection	3^b^	−
Stewart W; 1978	USA	Retrospective,single-center	1956–1976	1.677	116 (7)	−	811/866	−	−	BM detection	4^c^	−
Wang Y; 2021	China	Retrospective,population-based	2010–2016	1.317	186 (14)	67	−	−	−	Surgery	−	−
Wedin R; 2012	Sweden	Retrospective,single-center	1987–2007	31	31 (100)	59	−	−	−	Surgery	−	−
Wilson M; 2020	USA	Retrospective,single-center	2002–2017	463	198 (43)	−	295/168	I 47 (24), II 44 (22),III 78 (40), IV 27 (14)	BRAF 80 (49) NRAS 21 (21)	−	−	76^b^
Zekri J; 2017	UK	Retrospective,two-center	2000–2008	518	89 (17)	53	−	−	−	BM detection	4^b^	26^b^
Zhao Z; 2014	USA	Retrospective,multi-center	2005–2010	2.546	285 (11)	61	−	−	−	−	−	11^c^

Abbreviations: N = number of patients; yr = year; BMM = bone metastatic melanoma; M/F = male/female; BRAF = b-raf murine sarcoma viral oncogene homolog B1; NRAS = neuroblastoma ras viral oncogene homolog; T1 = time point measured until death; mo = months; USA = United States of America; UK = United Kingdom; BM = bone metastasis

a Overall survival is measured as the time from T1 to death.

b Median reported

c Mean reported

### 2.8 Data analysis

A quantitative meta-analysis for pooling incidences and hazard ratios was not performed due to the heterogeneity in patient populations, stages of disease, differences in treatment approaches and different diagnosis modalities for both bone metastases and SREs, inconsistent follow-up times, and variability in reporting the outcomes. Providing pooled incidences or hazard ratios with the data for different outcomes would result in hazard ratios with minimal clinical validity. Because all included studies were of comparable quality, scoring five or greater on the NOS scale, a quantitative summarization was provided by utilizing the range of incidence of bone metastases and SREs and listing risk factors for SREs and survival. We used Microsoft Excel Version 16.76 (Microsoft Inc, Redmond, WA, USA) to extract data using standardized forms and to create all figures and tables, Mendeley Desktop Version 1.19.8 (Mendeley Ltd, London, UK) as reference software, and Rayyan.ai (Rayyan Systems Inc, Boston, MA, USA) for article screening.

## 3 Results

### 3.1 Incidence of bone metastases

The most common modalities for determining bone metastases were as followed: imaging (62 %; 18/29), pathology reports (24 %; 7/29), and medical record notes such as progress notes (24 %; 7/29). Other modalities included autopsies, large databases, and histology reports. The average bone metastases-free interval ranged from four to 72 months. Incidence of bone metastases ranged from 2 % to 49 % in the 14 studies reporting the incidence. Gassenmaier et al. was the most extensive study, with 3,139 patients and a reported incidence of 30 % determined by imaging [17]. Wang et al. demonstrated a bone metastases incidence of 14 % in 1.317 patients with melanoma, as determined by pathology reports [29]. The location of bone metastases was described in 69 % (20/29) of studies, with 24 % (7/29) of studies focusing only on spinal metastases (Table 2).

**Table 2:** Incidences of bone metastases in melanoma patients (n = 29).

First author	Diagnosis method	BM free interval (mo)	Total Patients (N)	Patients with BM (N (%))	Numberof BM (N)	Location of BM (N (%))	Metastasis to other organs
Abdel-Rahman O	Pc (53.5 %), Clinical methods (45.5 %), unknown (1 %)	−	2.691	572 (21)	−	−	Yes (92 %)
Akslen L	Autopsy	−	62	37 (49)	−	−	Yes
Angela Y	Electronic database; x-ray	−	29	29 (100)	−	−	−
Berberich C	PET/CT	−	93	−	13	−	Yes
Bhanot K	Electronic database	−	2.646	91 (3)	−	Spine (100 %)	−
Bostel T	CT, MRI, BS	−	41	41 (100)	−	Spine (100 %)	Yes (98 %); lung (78 %); brain (34 %); other visceral (59 %); skin (44 %); lymphatic (70 %); other (29 %)
Brountzos E	MR, x-ray, CT, BS	37^a^^,c^	293	28 (10)	90	Axial/spine (67 %); Appendicular (33 %)	Yes
Brown L	CT, PET	−	264	24 (12)	−	−	−
Colman M	Pc	72^c^	130	130 (100)	258	Axial/spine (59 %); Appendicular (41 %)	Yes; lung (66 %); liver (46 %); brain (28 %); skin (35 %); other (37 %)
DeBoer D	Histologically confirmed	31^c^	283	14 (5)	−	Axial/spine (35 %); Appendicular (64 %)	No
Donaldson W	Electronic database	32^c^	15	15 (100)	>47	Spine (100 %)	Yes (100 %); lung (47 %); liver (27 %); brain (40 %); nodes (73 %); other (40 %)
Fon G	BS, biopsy, autopsy	43^c^	50	50 (100)	127	Axial/spine (68 %); Appendicular (32 %)	Yes; lung (36 %); brain (14 %); soft tissue (22 %); nodes (8 %); liver (16 %); intestine (4 %)
Gassenmaier M	MR, radiological images	−	3.139	431 (30)	−	−	Yes
Gerszten P	CT, MRI	−	28	28 (100)	36	Spine (100 %)	Yes
Gokaslan Z	X-ray, CT, MRI	21^d^	133	133 (100)	211	Spine (100 %)	Yes
Huang K	MR, radiological images, Pc	10^c^	11	11 (100)	31	Axial/spine (58 %); Appendicular (42 %)	Yes (100 %); liver (55 %); lung (45 %); nodes (55 %); brain (9 %); other (18 %)
Huttenlocher S	CT, MRI	n = 12 ≤ 15 mo; n = 15 > 15 mo^b^	27	27 (100)	−	Spine (100 %)	Yes
Krygier J	MR, radiological images, Pc	26^d^	37	37 (100)	41	Appendicular (100 %)	Yes
Mannavola F	Radiological images	−	305	305 (100)	−	Axial 165 (55 %), Appendicular 29 (10 %), both 106 (35 %)	Yes (97 %); without brain (73 %); with brain (24 %)
Rate W	MR, x-ray	−	77	26 (34)	39	Axial/spine (49 %), Appendicular 20 (51 %)	−
Sellin J	−	n = 42 < 12 mo;n = 22 > 12 mo	64	64 (100)	73	Spine (100 %)	Yes; lung (45 %); other visceral (61 %); soft-tissue (44 %)
Shankar G	−	−	18	18 (100)	−	−	−
Spiegel D	BS, x-ray, myelography, CT, MRI	4^c^	7.010	114 (2)	376	Axial/spine (100 %); Appendicular (63 %)	Yes
Stewart W	X-ray, BS	22^c^	1.677	116 (7)	190	Axial/spine (67 %); Appendicular (33 %)	−
Wang Y	Pc	−	1.317	186 (14)	−	−	Yes
Wedin R	Pc	−	31	31 (100)	34	Spine (35 %); Appendicular (65 %)	Yes
Wilson M	CT, MRI, and PET	−	463	198 (43)	−	Axial/spine (52 %); Appendicular (48 %)	Yes
Zekri J	Radiological images	2^d^	518	89 (17)	−	Axial/spine 47 (53 %); Appendicular 17 (19 %); both 25 (28 %)	−
Zhao Z	−	−	2.546	285 (11)	−	−	−

Abbreviations: BM = bone metastasis; mo = months; N = number; Pc = pathologically confirmed; x-ray = radiographs; PET = positron emission tomography; CT = computed tomography; MRI = magnetic resonance imaging; BS = bone scintigraphy; MR = medical records;

a For disease free interval before stage IV; ^b^Interval melanoma diagnosis to spinal cord compression; ^c^Mean recorded; ^d^Median recorded

### 3.2 Incidence of SREs

Of the 29 studies, 21 further investigated SREs, including pathological fracture (n = 12), spinal cord compression (n = 11), hypercalcemia (n = 4), radiation therapy (n = 17), and surgery to the bone (n = 16). Only one study, Zekri et al., specifically studied the incidence of SREs [4]. In total, 518 patients with melanoma were included of whom 17 % (83/518) developed bone metastases. Of these 83 patients with bone metastases, 71 % (n = 59) developed a cumulative total of 129 SREs (50 radiotherapy, 28 hypercalcemia, 20 bone fractures, 18 spinal cord compression and 13 surgery). The annual skeletal morbidity rate was 2.5 (95 % CI: 2.1–2.9), indicating that 2.5 SREs are reported per patient for every year of follow-up. A variety of first line treatments, including combination therapies, were used for patients with bone metastatic melanoma, with the most common being radiotherapy, followed by surgery, and chemotherapy (Table 3).

**Table 3:** Developments of bone pain and skeletal-related events in melanoma patients (n = 21).

First author	Bone pain (N)	Total SRE (N)	Pathological fracture (N)	Spinal cord compression (N)	Hypercalcemia (N)	Radiation therapy (N)	Surgery (N)	First line therapy for BM
Bostel T	15	65	24	−	−	41	−	RT (41)
Brountzos E	22	74	4	0	4	66	−	RT (66), RT/Chemo (31), Chemo (17)
Colman M	−	107	33	24	−	54	50	Sx (50), Chemo (66)
DeBoer D	−	14	−	−	−	9	5	Chemo (3), RT (2), RT/Chemo (4), Chemo/Sx (2), RT/Sx (1), RT/Chemo/Sx (2)
Donaldson W	−	14	1	−	−	9	4	Chemo (13), RT (9), Sx (4)
Fon G	−	74	74	−	−	−	−	−
Gerszten P	28	51	−	−	−	23	28	RT (23), Radiosurgery (6)
Gokaslan Z	−	116	−	7	−	−	−	−
Huang K	−	23	4	3	−	5	11	RT/Sx (5), Chemo/Sx (6), Immuno (3)
Huttenlocher S	−	27	−	−	−	27	−	RT (27)
Krygier J	4	71	20	−	−	10	41	RT (10), Sx (27)
Mannavola F	−	174	35	21	1	109	8	BDA (1 3 9); Immuno (1 7 4); Chemo (21); RT (1 0 9); Sx (8); Other (1 5 6)
Rate W	26	46	−	17	−	26	3	RT (26), Sx (3)
Sellin J	−	109	−	−	−	45	64	RT (45), Sx (64)
Shankar G	−	36	−	13	−	8	15	Sx (15), Radiosurgery (8)
Spiegel D	85	35	−	24	−	−	11	−
Stewart W	−	96	75	10	−	−	11	−
Wang Y	−	−	−	−	−	62	186	RT (62), Chemo (66), Sx (1 8 6)
Wedin R	−	−	19	12	−	14	31	RT (14), Chemo (15), Sx (31)
Wilson M	−	128	49	−	5	53	21	RT (53), Sx (21), BDA (40)
Zekri J	−	129	20	18	28	50	13	RT (45), Sx (12), BDA (27)

Abbreviations: SRE = skeletal-related events; BM = bone metastasis; RT = radiotherapy; Chemo = chemotherapy; Sx = surgery; Immuno = immunotherapy; BDA = bone directing agents.

### 3.3 Factors associated with developing bone metastases and/or SREs

Four studies investigated risk factors associated with the development of bone metastases [13], [19], [28], [31]. Risk factors found to be independently associated with developing bone metastases included: age, younger than 40 years; primary lesions that were ulcerated, deeper than Clark level II, thicker than 0.76 mm, or located on trunk or mucosal surfaces; presence of tumor-infiltrating lymphocytes; and elevated LDH levels (all p < 0.05). Other factors considered but not found to be associated with bone metastases were race, gender, laterality, T stage and N stage, mitotic index, and BRAF or NRAS mutations. Studies on the factors associated with SREs development were limited; one study by Mannavola et al. investigated this using univariate and multivariate analysis. The authors found that only the use of bone modifying agents such as bisphosphonates or denosumab before developing SREs was an independent prognostic factor that decreased the risk of developing SREs (OR 0.38, 95 % CI 0.2–0.72, p = 0.003) [23]. No clinical or tumor-related variables in this study correlated with SREs development (Table 4).

**Table 4:** Analysis of risk factors for developing bone metastasis and skeletal-related events in melanoma patients (n = 6).

First author	Type of analysis	Factors associated with risk of BM	Factors considered but not associated	Results
Abdel-Rahman O	Chi-square test	LDH, age	Race, gender, histology, laterality, T stage, N stage	Elevated LDH (p < 0.001) and age at diagnosis younger than 40 years (p = 0.044) were associated with higher risk of BM.
Gokaslan Z	Fisher's Exact test	−	−	Truncal primary tumors were more likely to metastasize to spine than primary melanomas from other cutaneous sites in females (p = 0.001).
Spiegel D	Chi-square test	Ulcerated primary lesions, lesions deeper than Clark level II, lesions thicker than 0.76 mm, and lesions that are located on the trunk or mucosal surfaces	Histologic type	Primary lesions that were ulcerated, deeper than Clark level II, thicker than 0.76 mm, or located on trunk or mucosal surfaces are associated with development of BM (p < 0.01).
Wilson M	Chi-square test	Stage at initial diagnosis, histology type, anatomic site, TIL	Gender, mitotic index, ulceration, *BRAF, NRAS*	Patients with BM had more advanced disease at the time of initial diagnosis (p = 0.005), primary tumor was more located in axial tissues (p < 0.001), had more TILs, and less likely to be nodular melanoma (p < 0.001).

First author	Type of analysis	Factors associated with risk of SRE	Factors considered but not associated	Results

Mannavola F	UA/MA	UA: localization of BM (axial vs. appendicular), use of bone targeting agents before SRE MA: use of bone targeting agents before SRE	Age, sex, histology, BRAF NRAS genotypes, time of diagnosis (synchronous vs. metachronous), number of BM, calcemia, LDH levels, systemic treatment	After both UA and MA, only the use of BDA prior to the development of SREs was confirmed as an independent prognostic factor (OR 0.38, 95 % CI 0.2–0.72, p = 0.003). Neither clinical variables nor tumor-related variables correlated with SREs.
Zekri J	−	−	−	The annual skeletal morbidity rate^a^ was 2.5 (95 % CI: 2.1, 2.9) i.e. 2.5 SREs are reported per patient for every year of follow-up.

Abbreviations: BM = bone metastasis; SRE = skeletal-related events; LDH = lactate dehydrogenase; mm = millimeters; TIL = tumor infiltrating lymphocytes; BRAF = V-Raf Murine Sarcoma Viral Oncogene Homolog B; NRAS = neuroblastoma RAS viral oncogene homolog; UA = univariate analysis; MA = multivariate analysis; BDA = bone directed agents; CI = confidence interval

^a^ Skeletal morbidity rate = number of SREs reported divided by the person years at risk

### 3.4 Survival analysis

Of the studies included in this review, 66 % (19/29) investigated survival outcomes in patients with bone metastatic melanoma (Table 5). Survival after the detection of bone metastases ranged between three and 13 months. Sellin et al. found the median survival after 64 consecutive patients undergoing surgery for spinal metastases to be six months (95 % CI, 2.7–28.7) [26]. Bostel et al. reported that only 37 % (15/41) of patients were alive six months after radiotherapy [35]. Patients who underwent immunotherapy and palliative radiotherapy showed an overall survival with a median of 17 months (95 % CI, 10.0–23.3) in 108 patients [23]. Factors found to be associated with survival included clinical (neurologic involvement, LDH and hemoglobin levels, Karnofsky performance status, ECOG), tumor-related (number and location of bone metastases, melanoma stage, presence of visceral/skin metastases, presence of pathological compression fracture of the spine), and treatment (systemic treatment) features. While some studies found no difference between axial and appendicular metastases on overall survival, DeBoer et al. found a survival advantage at 10 years in appendicular bone metastases (27 %) compared with axial metastases (0 %; p = 0.001) [39]. None of the studies provided an easy-to-use nomogram or algorithm to calculate survival percentages.

**Table 5:** Analysis of survival of melanoma patients (n = 19).

First author	Type of analysis	Factors associated with survival	Factors considered but not associated	Results
Bostel T	KM, UA	Number of bone metastases, visceral metastases, skin metastases, Karnofsky PS (70–90 % vs. 40–60 %)	Localization, brain met, lung met, other distant met, bisphoshponate use, orthopaedic corset use, chemotherapy	Five-year OS was 23 % and median BS 4 months (range 1–30). Only 37 % were alive at 6 months after RT.
Colman M	UA/MA	UA: number of total metastatic sites, pulmonary metastases, baseline cardiac disease, and presentation with spinal pathologic compression fractureMA: presentation with a pathologic compression fracture of the spine	−	Resection demonstrated a two-fold protective effect on OS after correcting for pathologic fractures. Median OS for the nonoperative, intralesional, and resection groups was 4.8, 5.1, and 11.8 months, respectively.
DeBoer D	KM	Appendicular vs. axial, staging of melanoma	−	Survival advantage (27 %) for the appendicular group at 10 years when compared with axial group (0 % at 3 years; p = 0.001).
Donaldson W	Descriptive	−	−	6 months (3 weeks-24 months) between diagnosis of spinal metastasis and death; 4 months (3 weeks-7 months) between neurological findings and survival; neurological symptoms with surgery 5 months; neurological symptoms without surgery 1 months. Average survival after initial diagnosis of melanoma 3 years (1–9 years)
Fon G	−	−	−	Mean survival time from bone metastasis diagnosis was 5 months
Gokaslan Z	−	−	−	Median survival after diagnosis of spinal mets was 4 months (1 week-43 months). In total, 66 % were dead at 6 months and 85 % at 1 year
Huang K	−	−	Axial vs. appendicular, age	Mean survival time from bone metastasis diagnosis was 6 months. There was no significant difference between axial vs. appendicular, and age. Men survived longer than women (7 vs. 4 months)
Huttenlocher S	UA/MA	UA: ECOG performance status, number of involved vertebrae, pre-RT ambulatory status, further bone mets, visceral mets, time to developing motor deficits MA: ECOG performance status, visceral mets	# of involved vertebrae, pre-RT ambulatory status, further BM, time developing motor deficits before RT	Survival rates at 6 months was 33 % and 12 months was 22 %.
Krygier J	KM; linear regression analysis		Number of metastatic disease at time of surgery, time of development of BM	The median survival from surgery was 9 months (range 1–135 months). The overall survival of 30 % at 12 months and 17 % at 24 months.
Mannavola F	UA/MA	UA: Sex, location, <5 BMs, LDH level, <3 metastatic sites, brain metastasis, systemic treatmentMA: <5 BMs, LDH level, <3 metastatic sites, systemic treatment	Age, histology, BRAF NRAS genotype, time of diagnosis, localization of BM, calcemia, use of BDA, SRE occurrence	Median OS from the diagnosis of bone metastasis was 11 months. Patients receiving immunotherapy and palliative radiotherapy showed the best overall survival (median = 17 months)
Rate W	−	−	−	Median survival with malignant melanoma metastatic to bone was 15 weeks and spinal cord compression was 13 weeks.
Sellin J	UA/MA	UA: Spinal metastasis after systemic metastasis, >2 spinal metastasis, progressive systemic disease, postoperative complications associated with worse survival; improved survival if only BM was present at surgery MA: progressive systemic disease, >2 spinal metastasis	Solitary spinal met, presence of lung met, presence of visceral met, presence of soft-tissue met	Involvement of ≥ 3 vertebral bodies and progressive systemic disease were associated with worse OS. Median OS was 5.7 months
Shankar G	UA	Preoperative immunotherapy, preoperative KPS	SINS, ESCC, BRAF, preoperative BRAF/MEK targeted therapy	Median PFS was 53 days and median OS was 100 days.
Spiegel D	KM; log-rank test	Number of additional metastatic disease at time of spinal metastases correlated with survival time	Distant skin metastasis nor those to lymph nodes, nor combination of the two; involvement of ≥ 1 spinal location	Median overall survival in patients with melanoma spinal metastasis was 86 days.
Stewart W	−	−	−	The mean survival time for all patients with skeletal lesions was 3.6 months. No method of treatment affected the prognosis once skeletal metastases occurred.
Wang Y	UA/MA	Tumor size (<2 or > 2 cm), additional metastases	Age, sex, RT, chemotherapy	The 1-, 3-, and 5-year OS rates of melanoma patients with bone metastases after surgery were 36 %, 15 %, and 10 %, respectively.
Wedin R	UA	Perioperative LDH < 8 vs. > 8, preoperative haemoglobin < 11.5 vs. > 11.5	−	Median survival after surgery was 1.9 months (range: 0–40); survival rate at 3-, 6-, 12-months after surgery was 39 %, 19 %, and 13 %, respectively.
Wilson M	UA/MA	UA: BMMA: BM, performance status, number of metastatic sites and elevated LDH	BDA	Median PFS was 3.9 months for melanoma patients with bone metastasis (n = 198). Median OS was 9.0 months
Zekri J	KM	**−**	−	Median survival of all patients from the diagnosis of BMs was 17 weeks with 8 % 1-year survival probability; median survival was 5.6 weeks after the first episode of hypercalcaemia.

Abbreviations: UA = univariate analysis; MA = multivariate analysis; KM = Kaplan-Meier; RT = radiotherapy; ECOG = Eastern Cooperative Oncology Group; LDH = lactate dehydrogenase; KPS = Karnofsky Performance Status; BDA = bone directed agents; SINS = Spinal Instability Neoplastic Score; ESCC = epidural spinal cord compression; OS = overall survival; BS = bone survival; PFS = progression-free survival; BM = bone metastasis

## 4 Discussion

Bone metastases and SREs pose a common problem in patients with melanoma, with incidence ranging up to 49 % and 66 %, respectively. While research has improved our understanding of the incidence of bone metastatic melanoma, there remains a substantial lack of knowledge in the literature, especially on the incidence of SREs and predictive factors for the development of bone metastases and SREs. A more comprehensive understanding through future studies investigating and reporting on predictive factors may help with patient and physician decision-making, improving outcomes in this patient population. Particularly, the establishment of easy-to-use nomograms or prediction models for developing bone metastases and subsequent SREs may guide both clinicians and patients in the clinical decision-making process. In addition, it can help identify patients at high risk for developing bone metastases and/or SREs, enabling the implementation of preventive measures and screening policies.

### 4.1 Incidence and characteristics of bone metastases

The cumulative incidence of bone metastases in melanoma patients was 13% in this systematic review, with the majority of diagnoses confirmed by imaging. The studies included in this review demonstrated more axial skeleton metastases sites, comparable to other studies examining skeletal sites in other primary solid tumors such as prostate and colorectal [40], [41]. Given the similar pattern of distribution across multiple primary tumors, this could be attributed to differences in local bone microenvironments between the appendicular and axial skeleton [42]. DeBoer et al. were unique in investigating patients with isolated bone metastasis [39]. Brountzos et al. meanwhile reported 3.7 % of patients with bone metastases as the initial and sole site of recurrence in a study of 28 patients [36]. While bone is a common site of metastasis, it is rarely an isolated event as it results from hematogenous spread, inherently making it likely that metastases also spreads to other organs. The proportion of patients with a BRAF and/or NRAS mutation was available in five studies. Apart from Brown et al., the incidence of BRAF mutations ranged between 49 % to 59 %, which is in concordance with that of all patients with melanoma [43]. While the frequency of NRAS mutation in the included studies was seemingly higher than in the general melanoma population, as reported in previous literature [44], this was not statistically significant due to the limited data available.

### 4.2 Risk factors of bone metastases

Elevated LDH, a younger age at diagnosis, and a melanoma site in the trunk/axial or mucosal areas were associated with a higher risk of developing bone metastases. While the prognostic influence of the primary melanoma site is less significant than that of histological factors and age, trunk melanomas have been significantly associated with advanced clinical stages and unfavorable survival rates and outcomes [45]. Its more aggressive characteristic could contribute to the metastatic spread to the bone. Potential reasons for a lower risk of bone metastases with increasing age could be angiogenic dormancy, the inability of micrometastases to grow due to a lack of vascularization, and age-related changes in the host tissue microenvironment [17]. Interestingly, other potential risk factors of bone metastases showed conflicting associations, including histology (e.g., ulceration) and cancer staging. This discrepancy could be attributed to the fact that while these features predict a poorer prognosis for patients with metastatic melanoma, they do not specifically predict metastasis to bone.

### 4.3 Characteristics of SREs

Zekri et al. reported an estimated average of 2.5 SREs per patient for every year of follow-up, which is consistent with previous reports worldwide [4], with an average range of 1.9 to 2.9 SREs per patient-year across all advanced cancers in various parts of Europe [46]. Radiation therapy and surgery were frequently used to manage SREs, serving as a common denominator in multiple studies investigating bone metastatic melanoma, where bone pain, pathological fracture, and spinal cord compression were often the indications for these interventions. While spinal cord compression and pathological fractures were recorded in approximately half of the studies related to SREs in this patient population, records on bone pain and hypercalcemia were sparse. Bone pain was a leading indication for intervention in all three studies that referenced SREs in patients with spinal metastatic melanoma [18], [28], [35]. This may suggest that the stability of vertebral bodies affected by bone metastases greatly influences patients’ quality of life and necessitates intervention for pain relief. Of the four studies that recorded hypercalcemia as an SRE, Zekri et al. reported the highest proportion of bone metastatic melanoma patients with hypercalcemia at approximately 23 %, demonstrating the imbalance between bone formation and resorption commonly seen in bone metastases from various primary tumors [4].

### 4.4 Risk factors of SREs

Mannavola et al. considered various melanoma and patient-associated factors for predicting SREs development in their patient cohort and identified the use of bone-directed agents as a protective factor against the development of SREs, resulting in a 62 % reduced risk of experiencing SREs and delayed time to their occurrence [23]. The decreased risk and delayed occurrence are consistent with findings in other primary tumor types, including breast, lung, and colorectal cancer [47], [48], [49]. The use of bone-directed agents such as bisphosphonates inhibits bone resorption, thereby decreasing serum calcium levels and delaying the pre-terminal SREs of hypercalcemia. Initiating earlier detection and treatment of bone metastases with bone-modifying agents could further amplify their potential benefits in reducing SREs occurrence. No other literature reviewed in this systematic study investigated risk factors of SREs. Further research focusing on risk factors for SREs is warranted to improve the quality of care.

### 4.5 Survivorship

The prognoses for patients with bone metastatic melanoma varied. Abdel et al. compared the survival between different metastatic sites within melanoma patients and observed worse survival outcomes for those who had single-site melanoma metastases at other sites, including skin, lymph nodes, and lungs [13]. The inferior survival is likely associated with the sequelae of the disease, with melanoma metastasis to bone occurring at a later stage in disease progression compared to other sites [50]. In studies prior to 2010, the average survival time ranged between four and six months [4], [15], [16], [19], [20]. Mannavola et al. included the most recent cohort of patients up until 2019 with a median overall survival from diagnosis of bone metastases of 10.7 months. Among these patients, those who underwent both immunotherapy and palliative therapy exhibited the highest survival rate, reaching 16.5 months [23]. These improvements in survival reflect the significant advancements in melanoma therapies that have been made in the past decade, with immunotherapies such as ipilimumab and targeted therapies such as vemurafenib offering promising options in treatment for patients with metastatic melanoma to the bone.

Survival rates of patients who underwent radiotherapy or surgical interventions were also reported. Bhanot et al. found that mortality following surgery for spinal metastatic melanoma was among the highest compared with other primary tumors, with a postoperative 90-day mortality of 51 % (46/91) [34]. It is important to emphasize that this does not reflect the survivability from the diagnosis of the primary tumor or bone metastases, as interventions might have occurred late in the disease process. In carefully selected patients, interventions can prolong survival. For example, Colman et al. reported a median survival of 11.8 months in those who underwent metastasectomy versus 4.8 months after nonoperative intervention, after controlling for patient- and disease-related variables [38].

Twelve studies investigated prognostic factors, where multiple bone metastases or the presence of visceral metastases were associated with decreased survival [21], [22], [23], [26], [27], [28], [29], [30], [31], [35], [38], [39]. Lower Karnofsky score and higher ECOG were associated with decreased survival [21], [27], [31], [35]. Surprisingly, Bostel et al. discovered that the presence of skin metastases was associated with increased survival in patients with spinal metastases after palliative radiotherapy [35]. This counterintuitive finding could be due to the cohort with skin metastases presented with better Karnofsky scores, lower rates of metastases to visceral organs, and more than one bone metastases. A multivariate analysis of this subgroup might have clarified this anomaly but was not conducted. In one retrospective review of 283 patients, patients with appendicular skeleton involvement survived three times as long as those with axial metastases, suggesting a survival advantage for this subset of patients [39]. Systemic treatment, such as targeted therapy or immune checkpoint inhibitor, demonstrated favorable survival compared to chemotherapy alone [23] or no immunotherapy at all [27], suggesting the utility and effectiveness of these innovative therapies. Other clinical variables independently associated with poor survival included elevated LDH levels [23], [30], [31], larger tumor size [29], and lower hemoglobin levels [30]. While several variables were explored across the studies, only a handful were consistently investigated, making it difficult to interpret the results.

### 4.6 Limitations and future recommendations

This systematic review has several limitations. First, the quality and comparability of the data were insufficient for a meta-analysis. However, we have systematically reviewed data according to the NOS guidelines to provide relevant information about current findings on bone metastatic melanoma and associated SREs. Second, we were limited to published material from the included studies, and other factors of interest, such as granular information regarding therapy, imaging methods used for diagnosis of bone metastases, and mutation type, were not readily available for review. Third, it was unclear whether patients were consecutively included in many of the studies, which introduces a risk of publication bias. Fourth, the study design and cohort differed among studies, making comparison difficult. Fifth, other relevant outcomes that could be considered more critical than survival, such as pain relief, preservation of physical function, quality of life, or adverse events following treatment for bone metastases and/or SREs, including surgical complications, hospitalizations, and reoperations, were not reported by the included studies. The findings of this review warrant well-designed retrospective and prospective studies that focus on the diagnosis, incidence and treatment outcomes of bone metastases and SREs in melanoma patients to better understand the current advancements and areas of improvement in treatments for this patient population. Providing user-friendly prediction models predicting not only survival but other important outcomes, such as quality of life, may guide and enhance the shared decision-making process of determining the optimal treatment. In addition, it can help start preventive measures and policies by identifying patients at high risk for developing bone metastases and subsequential detrimental SREs, such as early-on imaging for patients at high risk of developing bone metastases.

## 5 Conclusion

Advances in the treatment of melanoma have prolonged survival but have also resulted in increasing frequency of bone metastases ranging up to 49 %. The elevated incidence of bone metastases and the subsequent risk of SREs continue to pose challenges in patient outcomes and healthcare management. The current systematic review provides a comprehensive overview of the incidence, risk factors, and outcomes associated with bone metastases and SREs in patients with melanoma encompassing 29 studies. Yet, existing literature with high-quality data on especially predictive factors for metastatic melanoma to the bone is limited, and the heterogeneous design of studies poses a challenge in reaching firm conclusions. Future studies investigating and reporting on especially predictive factors, including granular information on treatment modalities, for bone metastases and SREs, could aid in guiding patient and physician decision-making, improving outcomes in this patient population.

## CRediT authorship contribution statement

**Michelle R. Shimizu:** Writing – original draft, Project administration, Formal analysis, Data curation. **Olaf N. van de Langerijt:** Writing – original draft, Formal analysis, Data curation. **Daniel Torres:** Data curation. **Tom de Groot:** Writing – review & editing. **Olivier Q. Groot:** Writing – review & editing, Supervision, Project administration, Conceptualization.

## Declaration of competing interest

The authors declare that they have no known competing financial interests or personal relationships that could have appeared to influence the work reported in this paper.

## Figures and Tables

**Table:** 

**Database**	PubMed	Embase	Cochrane Database
**Date**	July 23rd, 2023	July 23rd, 2023	July 23rd, 2023
**Number of hits**	3.286	1.620	56
**Search strategy**	(((“Melanoma”[Mesh] OR Melanomas OR “Malignant Melanoma*” OR “neoplasm*, skin” OR “tumor*, skin” OR “skin carcinoma*” OR “skin cancer” OR “cancer of the skin”) AND (“Bone metastases” OR “Bone metastasis” OR “Metastasis of Bone” OR “Metastases of Bone” OR “Skeletal metastases” OR “Skeletal metastasis” OR “Skeletal-related event*” OR “Skeletal complication*” OR “Impending fracture*” OR “Pathological fracture*” OR Hypercalcemia OR “Cord compression” OR “radiation to bone” OR “Surgery to bone” OR “Orthopedic surgery” OR “Orthopaedic surgery”))) OR (((“Melanoma”[Mesh] OR Melanomas OR “Malignant Melanoma*” OR “neoplasm*, skin” OR “tumor*, skin” OR “skin carcinoma*” OR “skin cancer” OR “cancer of the skin”) AND (“spinal metastases” OR “spinal metastasis” OR “Metastasis of spine” OR “metastases of spine” OR “spine metastases” OR “spine metastasis” OR “vertebral metastasis ” OR “ vertebral metastases” OR “metastatic to the spine” OR “metastatic to bone” OR “metastatic to the spine” OR “metastatic vertebral melanoma” OR “vertebral column melanoma” OR “vertebral melanoma”))) AND (1900/1/1:2023/7/23[pdat])	((‘melanoma’/exp OR ‘melanoma’ OR melanomas OR ‘malignant melanoma*’ OR ‘neoplasm*, skin’ OR ‘tumor*, skin’ OR ‘skin carcinoma*’ OR ‘skin cancer’/exp OR ‘skin cancer’ OR ‘cancer of the skin’/exp OR ‘cancer of the skin’) AND (‘bone metastases’ OR ‘bone metastasis’ OR ‘metastasis of bone’ OR ‘metastases of bone’ OR ‘skeletal metastases’ OR ‘skeletal metastasis’ OR ‘skeletal-related event*’ OR ‘skeletal complication*’ OR ‘impending fracture*’ OR ‘pathological fracture*’ OR hypercalcemia OR ‘cord compression*’ OR ‘radiation to bone’ OR ‘surgery to bone’ OR ‘orthopedic surgery’ OR ‘orthopaedic surgery’) OR ((‘melanoma’/exp OR ‘melanoma’ OR melanomas OR ‘malignant melanoma*’ OR ‘neoplasm*, skin’ OR ‘tumor*, skin’ OR ‘skin carcinoma*’ OR ‘skin cancer’/exp OR ‘skin cancer’ OR ‘cancer of the skin’/exp OR ‘cancer of the skin’) AND (‘spinal metastases’ OR ‘spinal metastasis’ OR ‘metastasis of spine’ OR ‘metastases of spine’ OR ‘spine metastases’ OR ‘spine metastasis’ OR ‘vertebral metastasis’OR ‘vertebral metastases’ OR ‘metastatic to the spine’ OR ‘metastatic to bone’ OR ‘metastatic vertebral melanoma’ OR ‘vertebral column melanoma’ OR ‘vertebral melanoma’))) AND [embase]/lim NOT [medline]/lim AND [01–01–1900]/sd NOT [24–07–2023]/sd	((Melanoma OR “Malignant Melanoma” OR “skin carcinoma” OR “skin cancer” OR “cancer of the skin”):ti,ab,kw AND (“Bone metastases” OR “Bone metastasis” OR “Metastasis of Bone” OR “Metastases of Bone” OR “Skeletal metastases” OR “Skeletal metastasis” OR “Skeletal-related event” OR “Skeletal complication” OR “Impending fracture” OR “Pathological fracture” OR Hypercalcemia OR “Cord compression” OR “radiation to bone” OR “Surgery to bone” OR “Orthopedic surgery” OR “Orthopaedic surgery”)) OR ((Melanoma OR “Malignant Melanoma” OR “skin carcinoma” OR “skin cancer” OR “cancer of the skin”):ti,ab,kw AND (“spinal metastases” OR “spinal metastasis” OR “Metastasis of spine” OR “metastases of spine” OR “spine metastases” OR “spine metastasis” OR “vertebral metastasis “ OR “vertebral metastases” OR “metastatic to the spine” OR “metastatic to bone” OR “metastatic to the spine” OR “metastatic vertebral melanoma” OR “vertebral column melanoma” OR “vertebral melanoma”))(Word variations have been searched)

**Table:** 

**Study**	**Reason for exclusion**
Conway JW, Rawson RV, Lo S, Ahmed T, Vergara IA, Gide TN, et al. Unveiling the tumor immune microenvironment of organ-specific melanoma metastatic sites. J Immunother Cancer [Internet]. 2022;10(9). Available from: http://dx.https://doi.org/10.1136/jitc-2022–004884	Internal validation
Herbert SH, Solin LJ, Rate WR, Schultz DJ, Hanks GE. The effect of palliative radiation therapy on epidural compression due to metastatic malignant melanoma. Cancer [Internet]. 1991;67(10):2472–6. Available from: http://dx.https://doi.org/10.1002/1097–0142(19910515)67:10 < 2472::aid-cncr2820671014 > 3.0.co;2-v	Internal validation
Schoenewolf NL, Belloni B, Simcock M, Tonolla S, Vogt P, Scherrer E, et al. Clinical implications of distinct metastasizing preferences of different melanoma subtypes. Eur J Dermatol [Internet]. 2014;24(2):236–41. Available from: http://dx.https://doi.org/10.1684/ejd.2014.2292	Internal validation
Amit M, Tam S, Abdelmeguid AS, Kupferman ME, Su SY, Raza SM, et al. Patterns of treatment failure in patients with sinonasal mucosal melanoma. Ann Surg Oncol [Internet]. 2018;25(6):1723–9. Available from: http://dx.https://doi.org/10.1245/s10434-018–6465-y	Focus on sinonasal mucosal melanoma
Cohn-Cedermark G, Månsson-Brahme E, Rutqvist LE, Larsson O, Singnomklao T, Ringborg U. Metastatic patterns, clinical outcome, and malignant phenotype in malignant cutaneous melanoma. Acta Oncol [Internet]. 1999;38(5):549–57. Available from: http://dx.https://doi.org/10.1080/028418699431122	Recurrent metastatic disease
Lau D, Than KD, La Marca F, Park P. Independent predictors for local recurrence following surgery for spinal metastasis. Acta Neurochir (Wien) [Internet]. 2014;156(2):277–82. Available from: http://dx.https://doi.org/10.1007/s00701-013–1973-9	Recurrent metastatic disease
Zeng KL, Sahgal A, Husain ZA, Myrehaug S, Tseng C-L, Detsky J, et al. Local control and patterns of failure for “Radioresistant” spinal metastases following stereotactic body radiotherapy compared to a “Radiosensitive” reference. J Neurooncol [Internet]. 2021;152(1):173–82. Available from: http://dx.https://doi.org/10.1007/s11060-020–03691-6	No focus on melanoma bone metastases
Vestergaard P, Rejnmark L, Mosekilde L. Fracture risk in patients with different types of cancer. Acta Oncol [Internet]. 2009;48(1):105–15. Available from: http://dx.https://doi.org/10.1080/02841860802167490	No focus on melanoma bone metastases
Conway JW, Rawson RV, Lo S, Ahmed T, Vergara IA, Gide TN, et al. Unveiling the tumor immune microenvironment of organ-specific melanoma metastatic sites. J Immunother Cancer [Internet]. 2022;10(9). Available from: http://dx.https://doi.org/10.1136/jitc-2022–004884	No focus on melanoma bone metastases

**Table:** 

Study	Cohort Star Template				
	**Selection**	**Comparability**	**Exposure**	**NOS score**	**Risk of Bias**
Abdel-Rahman O (1)	***		***	6	Low
Akslen L (2)	**		***	5	Unclear
Angela Y (3)	**	*	***	6	Low
Berberich C (4)	****		***	7	Low
Bhanot K (5)	***	**	***	8	Low
Bostel T (6)	****	*	***	8	Low
Brountzos E (7)	***		***	6	Low
Brown L (8)	***	**	*	6	Low
Colman M (9)	****	**	***	9	Low
DeBoer D (10)	**	**	**	6	Low
Donaldson W (11)	**		***	5	Unclear
Fon G (12)	**		***	5	Unclear
Gassenmaier M (13)	****	**	***	9	Low
Gerszten P (14)	***		***	6	Low
Gokaslan Z (15)	**		***	5	Unclear
Huang K (16)	**		***	5	Unclear
Huttenlocher S (17)	***		***	6	Low
Krygier J (18)	**		***	5	Unclear
Mannavola F (19)	****	**	***	9	Low
Rate W (20)	***	**	***	8	Low
Sellin J (21)	***	*	**	6	Low
Shankar G (22)	***		**	5	Unclear
Spiegel D (23)	****		***	7	Low
Stewart W (24)	***		***	6	Low
Wang Y (25)	****	**	**	8	Low
Wedin R (26)	**		***	5	Unclear
Wilson M (27)	****	**	**	8	Low
Zekri J (28)	***		**	5	Unclear
Zhao Z (29)	***		**	5	Unclear

**Figure:**
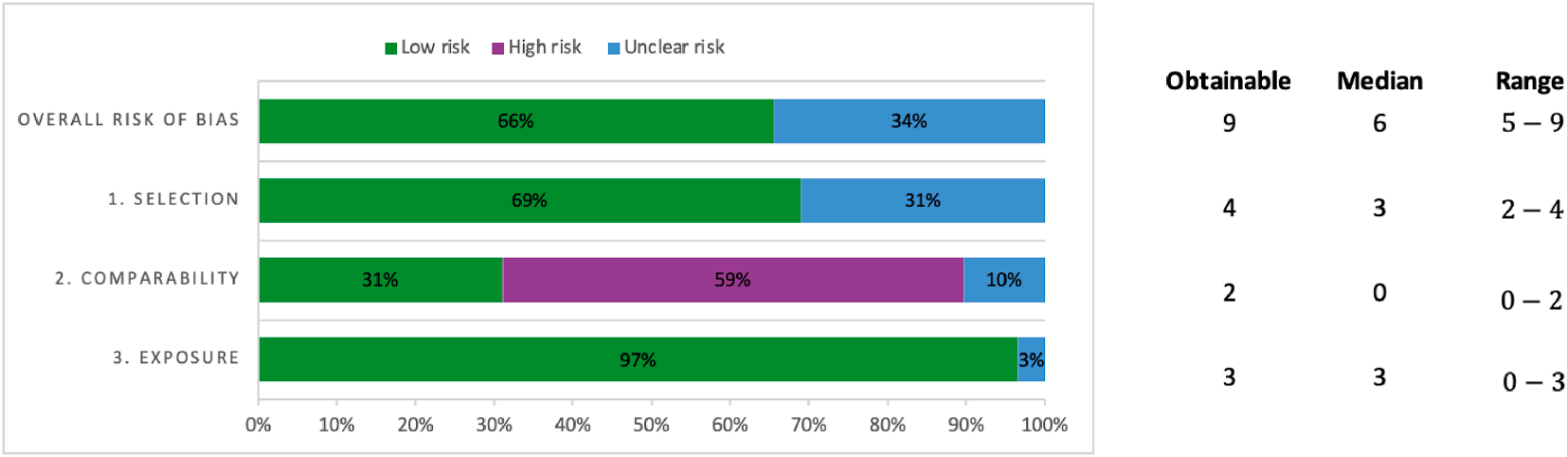


**Table:** 

Section and Topic	Item #	Checklist item	Location where item is reported
**TITLE**			
Title	1	Identify the report as a systematic review.	P.1
**ABSTRACT**			
Abstract	2	See the PRISMA 2020 for Abstracts checklist.	P.2–3
**INTRODUCTION**			
Rationale	3	Describe the rationale for the review in the context of existing knowledge.	P. 4
Objectives	4	Provide an explicit statement of the objective(s) or question(s) the review addresses.	P. 4–5
**METHODS**			
Eligibility criteria	5	Specify the inclusion and exclusion criteria for the review and how studies were grouped for the syntheses.	P. 6
Information sources	6	Specify all databases, registers, websites, organisations, reference lists and other sources searched or consulted to identify studies. Specify the date when each source was last searched or consulted.	P. 5
Search strategy	7	Present the full search strategies for all databases, registers and websites, including any filters and limits used.	Appendix A
Selection process	8	Specify the methods used to decide whether a study met the inclusion criteria of the review, including how many reviewers screened each record and each report retrieved, whether they worked independently, and if applicable, details of automation tools used in the process.	P. 5–6
Data collection process	9	Specify the methods used to collect data from reports, including how many reviewers collected data from each report, whether they worked independently, any processes for obtaining or confirming data from study investigators, and if applicable, details of automation tools used in the process.	P. 7
Data items	10a	List and define all outcomes for which data were sought. Specify whether all results that were compatible with each outcome domain in each study were sought (e.g. for all measures, time points, analyses), and if not, the methods used to decide which results to collect.	P. 7
	10b	List and define all other variables for which data were sought (e.g. participant and intervention characteristics, funding sources). Describe any assumptions made about any missing or unclear information.	P. 7
Study risk of bias assessment	11	Specify the methods used to assess risk of bias in the included studies, including details of the tool(s) used, how many reviewers assessed each study and whether they worked independently, and if applicable, details of automation tools used in the process.	P. 6
Effect measures	12	Specify for each outcome the effect measure(s) (e.g. risk ratio, mean difference) used in the synthesis or presentation of results.	P. 8
Synthesis methods	13a	Describe the processes used to decide which studies were eligible for each synthesis (e.g. tabulating the study intervention characteristics and comparing against the planned groups for each synthesis (item #5)).	P. 6
	13b	Describe any methods required to prepare the data for presentation or synthesis, such as handling of missing summary statistics, or data conversions.	NA
	13c	Describe any methods used to tabulate or visually display results of individual studies and syntheses.	NA
	13d	Describe any methods used to synthesize results and provide a rationale for the choice(s). If *meta*-analysis was performed, describe the model(s), method(s) to identify the presence and extent of statistical heterogeneity, and software package(s) used.	P. 8
	13e	Describe any methods used to explore possible causes of heterogeneity among study results (e.g. subgroup analysis, *meta*-regression).	NA
	13f	Describe any sensitivity analyses conducted to assess robustness of the synthesized results.	NA
Reporting bias assessment	14	Describe any methods used to assess risk of bias due to missing results in a synthesis (arising from reporting biases).	NA
Certainty assessment	15	Describe any methods used to assess certainty (or confidence) in the body of evidence for an outcome.	NA
**RESULTS**			
Study selection	16a	Describe the results of the search and selection process, from the number of records identified in the search to the number of studies included in the review, ideally using a flow diagram.	Fig. 1; P. 7
	16b	Cite studies that might appear to meet the inclusion criteria, but which were excluded, and explain why they were excluded.	Appendix B
Study characteristics	17	Cite each included study and present its characteristics.	P. 7; table 1
Risk of bias in studies	18	Present assessments of risk of bias for each included study.	Appendix C
Results of individual studies	19	For all outcomes, present, for each study: (a) summary statistics for each group (where appropriate) and (b) an effect estimate and its precision (e.g. confidence/credible interval), ideally using structured tables or plots.	NA
Results of syntheses	20a	For each synthesis, briefly summarise the characteristics and risk of bias among contributing studies.	NA
	20b	Present results of all statistical syntheses conducted. If *meta*-analysis was done, present for each the summary estimate and its precision (e.g. confidence/credible interval) and measures of statistical heterogeneity. If comparing groups, describe the direction of the effect.	NA
	20c	Present results of all investigations of possible causes of heterogeneity among study results.	NA
	20d	Present results of all sensitivity analyses conducted to assess the robustness of the synthesized results.	NA
Reporting biases	21	Present assessments of risk of bias due to missing results (arising from reporting biases) for each synthesis assessed.	NA
Certainty of evidence	22	Present assessments of certainty (or confidence) in the body of evidence for each outcome assessed.	NA
**DISCUSSION**			
Discussion	23a	Provide a general interpretation of the results in the context of other evidence.	P. 11–16
	23b	Discuss any limitations of the evidence included in the review.	P. 17
	23c	Discuss any limitations of the review processes used.	P. 17
	23d	Discuss implications of the results for practice, policy, and future research.	P. 17
**OTHER INFORMATION**			
Registration and protocol	24a	Provide registration information for the review, including register name and registration number, or state that the review was not registered.	P. 5
	24b	Indicate where the review protocol can be accessed, or state that a protocol was not prepared.	P. 5
	24c	Describe and explain any amendments to information provided at registration or in the protocol.	NA
Support	25	Describe sources of financial or non-financial support for the review, and the role of the funders or sponsors in the review.	P. 1
Declaration of competing interests	26	Declare any competing interests of review authors.	NA
Availability of data, code and other materials	27	Report which of the following are publicly available and where they can be found: template data collection forms; data extracted from included studies; data used for all analyses; analytic code; any other materials used in the review.	NA

## Appendix B**.** Overview of excluded studies after full-text assessment with reason for exclusion (n = 9)

Appendix C**.** Quality assessment according to the Newcastle-Ottawa Scale (NOS)Appendix D. Graphic representation of quality assessment with “low, high and unclear risk of bias” according to the Newcastle-Ottawa Scale (NOS)

*From:* Page MJ, McKenzie JE, Bossuyt PM, Boutron I, Hoffmann TC, Mulrow CD, et al. The PRISMA 2020 statement: an updated guideline for reporting systematic reviews. BMJ 2021;372:n71. https://doi.org/10.1136/bmj.n71.

Studies after duplicates removed(n = 4,641).
